# Influences of radiation on carp from farm ponds in Fukushima

**DOI:** 10.1093/jrr/rrv076

**Published:** 2015-12-13

**Authors:** Yuzuru Suzuki

**Affiliations:** Fisheries Laboratory, The University of Tokyo, Bentenjima, Maisaka, Hamamatsu-City, Shizuoka 431-0214, Japan

## Abstract

A massive release of artificial radionuclides from the Fukushima Dai-ichi Nuclear Power Plant caused radioactive contamination of farms as well as of aquatic products. Carp in small ponds in the highly radiocontaminated area of Iitate Village, Fukushima Prefecture, have been confined to the ponds since the accident, and it is thought that the carp may have suffered health issues as a result. Therefore, I investigated the health condition of the carp in order to elucidate the effects of radiation.

Blood neutrophil, monocyte and lymphocyte counts in the carp from three ponds in Fukushima were lower than those in carp from a non-polluted pond in Tochigi Prefecture. Histological observations indicated abnormal hyperplasia of macrophages in the spleen, kidney, liver and pancreas of carp in Fukushima. Although there are likely to have been deleterious effects on carp health due to the radiation in Fukushima, this has not yet been confirmed because only one control pond was available for comparison, and I was not able to find any symptoms in the carp that correlated with internal cesium concentration. Further research is now being conducted to investigate the effects of radiation on carp.

## INTRODUCTION

The Great East Japan Earthquake on 11 March 2011 caused an explosion at the Fukushima Dai-ichi Nuclear Power Plant (NPP), with a massive release of artificial radionuclides into the environment. The pollution spread widely and caused radioactive contamination of farms as well as of aquatic products. The Japanese government has reported that radioactive cesium concentrations of some wild freshwater fish species in Fukushima Prefecture still exceed the limit, i.e. 100 Bq/kg, whereas the radioactive cesium concentrations in marine fish species are fairly low and are in a declining trend [1]. The freshwater fish species have been internally exposed to radiation since the accident, which may have affected their health.

There are data indicating that, relative to other vertebrates, fish are not as susceptible to radiation; lethal doses of irradiation are generally higher in fish than in avian and mammalian species [2] (although this is based on observations on the acute effects of high-dose radiation). After the Chernobyl accident, a great effort was made to investigate the chronic effects of low-dose radiation on wildlife. However, little attention was paid to aquatic species, except for a brief description in a report by Ryabov [3], who observed serious disturbances in gonad morphology in the predatory fish, pike and perch. The chronic effects of low-level radiation on fish health have thus remained unknown.

In view of this, I have commenced an investigation of the effects of radiation on the health condition of the carp that live in small ponds in the highly radiocontaminated areas of Iitate Village in Fukushima Prefecture. Although I was only able to set one sampling point in an unpolluted control pond, the results suggest that low-dose chronic exposure has had considerable effect, as discussed below.

## OUTLINE OF THE OBSERVATIONS IN 2013

### Sampling

**Fig. 1:**
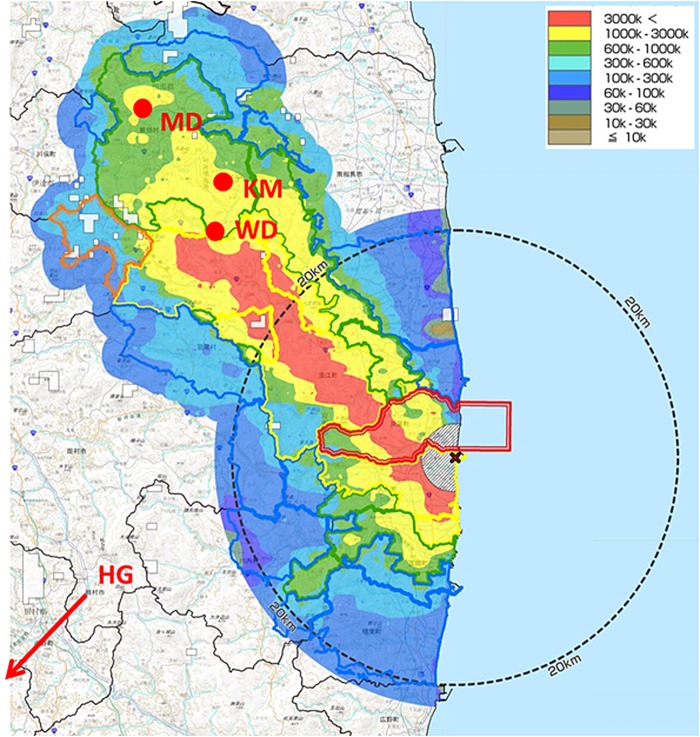
Location of the sampling points in Fukushima Prefecture, plotted on the map of the fallout from the Fukushima Daiichi Nuclear Power Plant.

Samplings were performed on 4 August 2013 at three farm ponds in polluted areas of Iitate Village, Fukushima Prefecture (MD, KM, WD), and on 3 August at a non-polluted control pond in Haga Town, Tochigi Prefecture (HG). In other proposed control ponds in Odawara (Kanagawa Prefecture) and Toyohashi (Aichi Prefecture), carp could not be captured. The sampling spots were plotted on a map of the surface fall-out of cesium following the NPP accident (Fig. 1). All the ponds have been kept in fairly natural conditions and the fish there have not been fed artificial food.

**Fig. 2.**
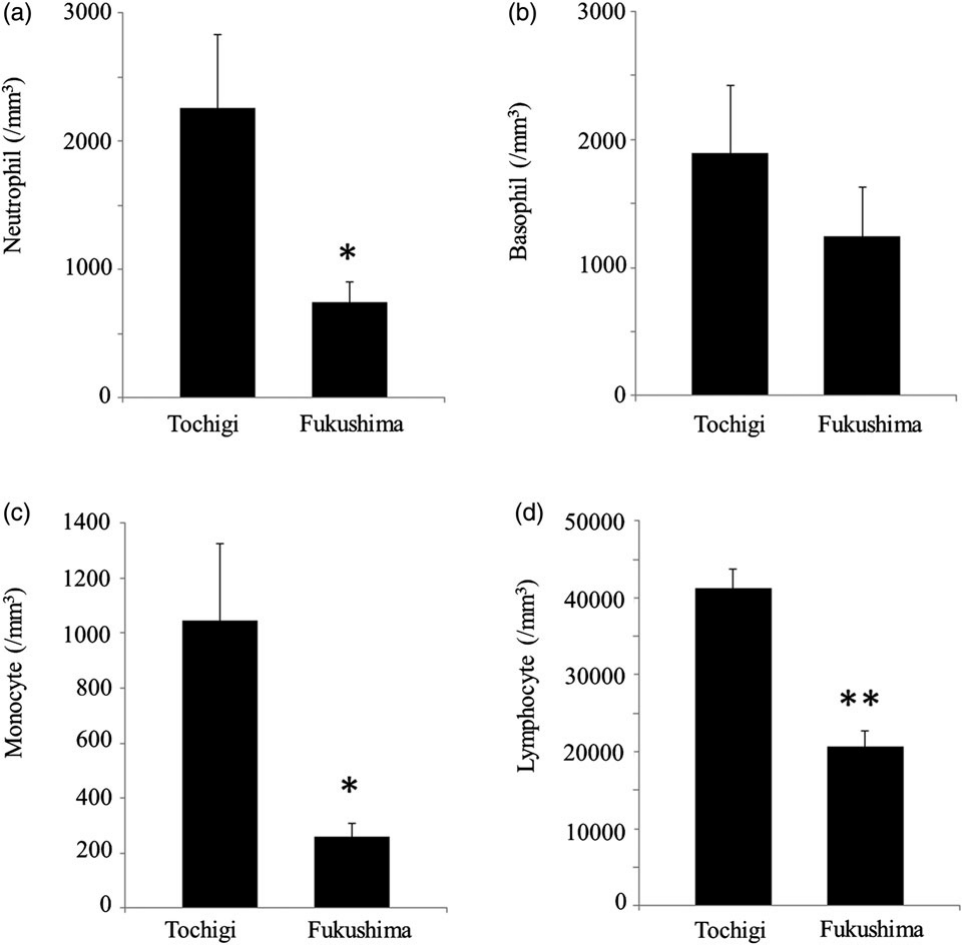
Comparisons of leukocyte counts in carp from polluted ponds in Fukushima and from a non-polluted pond in Tochigi. Each graph shows one of four types of leukocytes: (**a**) neutrophils, (**b**) basophils, (**c**) monocytes and (**d**) lymphocytes. Each bar indicates ± SD. **P < 0.01, *P < 0.05.

Fish caught by angling were anesthetized with 200-ppm quinaldine sulfate, and blood was taken from a caudal vessel with a heparinized syringe and needle. Erythrocytes (red blood cells) were counted using a hemocytometer under a microscope, and smear preparations were made for the leukocytes (white blood cells) count.

Tissues of spleen, kidney, head kidney, and hepatopancreas were fixed with 10% formalin, thus preserving samples for histological observation.

Muscles were minced for measurement of radioactive cesium concentration. Water and mud samples of the ponds were also taken for radioactive cesium analysis.

### Radioactivity

Radioactive cesium concentrations were measured by the Radioactivity Monitoring Center for Citizens (Chikurinsha), using a Germanium semiconductor detector, which was supplied by Association pour le Contrôle de la Radioactivité dans L’Ouest, an NGO measuring radioactivity and editing a quarterly review in French. The data collector was an ORION digital collecting system by Itech Instruments. The software for the analysis was Inter Winner by Itech Instruments.

The cesium concentrations are summarized in Table 1. Dried mud from the bottom of the ponds in Fukushima showed extremely high radioactivity, whereas the equivalent samples from Tochigi had low radioactivity. On the other hand, the water from each pond showed no cesium contamination. In carp muscles, high cesium concentration was detected in individuals from ponds KM and WD, which had highly contaminated mud at the bottom. Carp in pond MD also showed high cesium concentration, although the levels were lower than those in carp from pond KM and WD, in accordance with the mud cesium concentrations. In contrast, pond HG in Tochigi showed low contamination, indicating the pond was suitable as a control. The cesium concentration in carp muscles from pond HG was very low compared with those from ponds KM, WD and MD. These results indicate that the cesium in carp muscles would be derived from the mud at the bottom of the ponds, although it is not clear whether the route is by ingestion of the mud or by biological accumulation via the food web.

**Table 1.** Mean radioactive Cesium concentrations in dried mud from the bottom of the ponds and in carp muscle at each sampling point

Sampling point	WT (°C)	Cesium in mud (Bq/kg)			Cesium in carp muscle (Bq/kg)			
		^134^Cs	^137^Cs	Cs total	*n*	^134^Cs	^137^Cs	Cs total
KM13	23	22 000	53 000	75 000	4	1540	3493	5033 ± 1157
WD13	18	9100	21 000	30 100	5	1818	4168	5986 ± 2619
MD13	24	3600	8200	11 800	5	402	921	1323 ± 731
HG13	26	36	55	91	4	3	10	13 ± 6

WT = water temperature, *n* = number of carp individuals. Total Cs in carp muscle is expressed as mean ± SD.

### Leukocyte count

Smear preparations were stained with May–Grünwald Giemsa stain. As erythrocyte was already estimated, leukocyte count was calculated by the ration leukocytes to erythrocyte by the observations of smear preparations. Thereafter each count of leukocytes, i.e., neutrophil, basophil, lymphocyte and monocyte, was calculated by the ratio of these types of leukocyte.

**Fig. 3.**
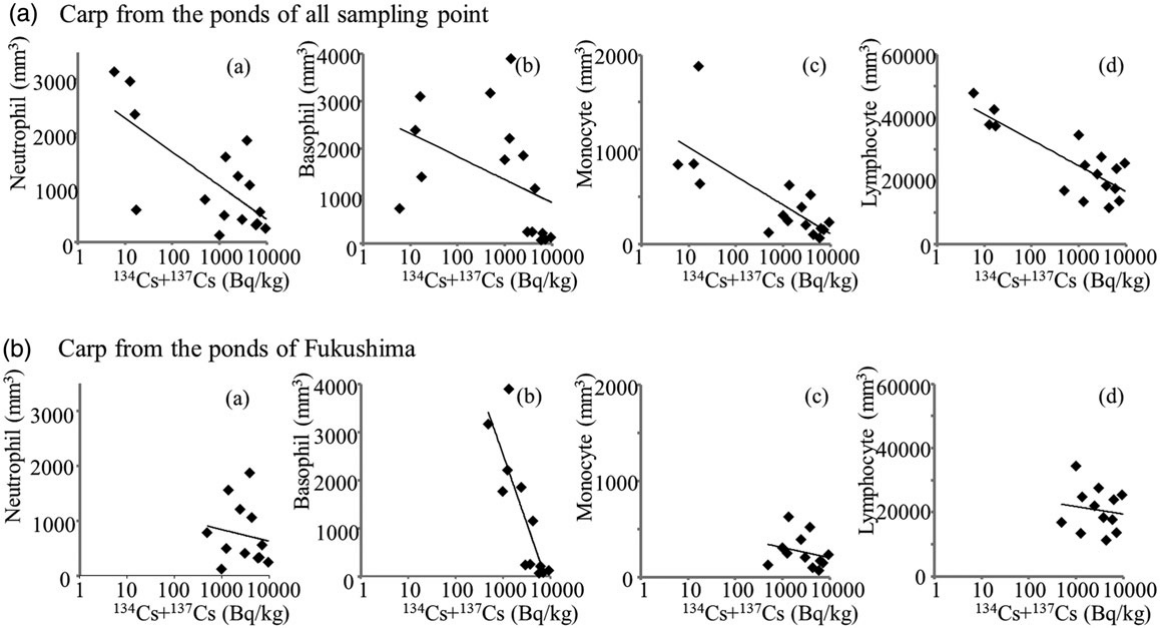
Relationships between muscle Cesium concentrations and leukocyte counts in carp (**a**) from the ponds of all sampling points, and (**b**) from the ponds of Fukushima. Each graph shows one of four types of leukocytes: (a) neutrophils, (b) basophils, (c) monocytes and (d) lymphocytes.

Figure 2 shows the comparisons of leukocyte counts for the carp in Fukushima and Tochigi. The carp in Fukushima had fewer leukocytes of all types than those in Tochigi, although the difference in basophil numbers was not significant. These observations show a possible inhibitory effect of chronic radiation on hemopoietic activities in the carp in Fukushima. Figure 3 shows the relationships between radioactive cesium concentrations in carp muscles and each leukocyte count. These figures seem to indicate that leukocyte counts correlate negatively with cesium concentration. However, this relationship might be due to differences in the sampling locations of Fukushima and Tochigi. In fact, when the data of Tochigi were omitted, the counts of leukocytes except basophil did not significantly show negative correlations with cesium concentrations. Lack of data between 10 and 1000 Bq/kg was a fatal defect for certification of the relationship. Reports on the human children in Ukraine, and on the juvenile Japanese monkeys in Fukushima, where such levels of cesium concentration showed clear effects on the blood cells in a dose–response manner [4], also indicate the importance of a study of low-level radiation.

### Histology

Tissues were dehydrated in ethanol and xylene, and embedded in paraffin. Sections were prepared (thickness 5 μm) and stained with hematoxylin and eosin for microscopy.

**Fig. 4.**
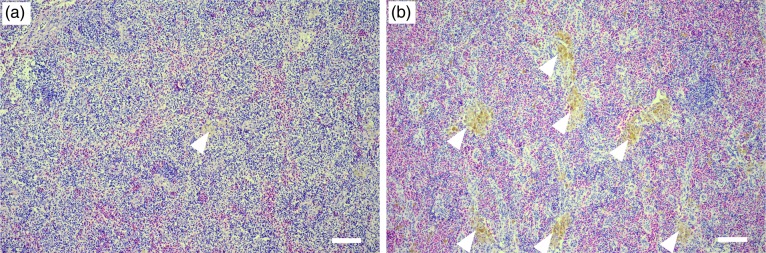
Spleen sections of carp in Tochigi (**a**) and Fukushima (**b**). Many specimens of carp in Fukushima showed hyperplasia of macrophage aggregates (arrowheads). Scale bars = 100 μm.

Hyperplasia of macrophage aggregates was observed in the carp from Fukushima, but not Tochigi (Fig. 4). Fish spleen and kidney usually have macrophage aggregates termed melano-macrophage centers (MMCs) [5], as shown in Fig. 4a. However, the size and the number of MMCs in the carp from Fukushima were excessive, as shown in Fig. 4b. In addition, these fish have MMC-like structure, even in liver and pancreas, where macrophages are rare in carp. The macrophages are functioning to eliminate invading foreign materials such as bacteria, as well as virus-infected or self-damaged cells. Abnormal macrophage proliferation suggests the increase in cell damage caused by internal exposure to radiation, although the extent of abnormality did not increase/decrease according to the cesium concentration.

## ONGOING RESEARCH PROJECT IN 2014

Although the results of the investigation in 2013 showed a clear decrease in leukocyte counts and histological abnormality in the carp from Iitate Village, Fukushima Prefecture, it is not yet certain that these changes were caused by the radiation. It is possible that I was observing a difference due to locality, Fukushima versus Tochigi, because I had only one control pond. In addition, I couldn't obtain samples of between 10 and 1000 Bq/kg; samples within this range will be very important for investigating the relationship between radiation levels and leukocyte counts. The project in 2014 was planned to solve these questions. My collaborator and I set two control ponds, one in Tochigi and the other one in Gunma Prefecture. Fish samplings were performed in fairly low-level contaminated areas, Minamisoma City and Kawauchi Village, Fukushima Prefecture. Tissue samples fixed with RNA were later also taken, and the expression analyses will be performed by a collaborator.

Unfortunately, we failed to catch carp in Gunma. In addition, the cesium levels of carp from Minamisoma were found to be much higher than expected, as shown in Table 2. The other results will be reported at a later date, when we finish the analyses of all samples.

**Table 2.** Mean radioactive Cesium concentrations in carp muscle from sampling points in 2014 and 2013

Sampling Point	Cesium in carp muscle (Bq/kg)			
	*n*	^134^Cs	^137^Cs	Cs total
Sampling in 2014				
NG14	5	306 ± 122	1004 ± 410	1310 ± 532
MS14	4	243 ± 69	815 ± 226	1058 ± 294
MD14	5	100 ± 56	328 ± 160	438 ± 214
KW14	5	20.6 ± 7.8	69.4 ± 34.2	90 ± 41.7
HG14	5		6.3 ± 1.1	6.3 ± 1.1
Sampling in 2013				
MD13	5	402 ± 198	922 ± 456	1324 ± 653
HG13	4	2.9 ± 2.9	9.7 ± 2.2	12.6 ± 4.8

*n* = number of carp individuals. Total Cs in carp muscle is expressed as mean ± SD.

## CONCLUSION

Low-level chronic exposure to radiation may damage the health of fish, even though the radioactive cesium contents in the muscles of carp from Fukushima were much lower than the lethal level in fish [1]. Although fish are believed to be less susceptible to radiation than many other vertebrates [1], this does not necessarily mean that fish are healthy under the long term low dose radiation condition. Continued investigations in Fukushima are needed to clarify further the effects of low-level radiation.

## FUNDING

Funding to pay the Open Access publication charges for this special issue was provided by the Grant-in-Aid from the Japan Society for the Promotion of Science (JSPS) [KAKENHI Grant No. 26253022].
